# Management of a Pregnancy Complicated by Pompe Disease

**DOI:** 10.1155/2012/137861

**Published:** 2012-12-10

**Authors:** Jennifer Weida, B. E. Hainline, C. Bodkin, M. K. Williams

**Affiliations:** ^1^Division of Clinical and Biochemical Genetics, Department of Medical and Molecular Genetics, Indiana University School of Medicine, 975 West Walnut Street, IB 130, Indianapolis, IN 46202, USA; ^2^Department of Neurology, Indiana University School of Medicine, 975 West Walnut Street, IB 130, Indianapolis, IN 46202, USA; ^3^Division of Maternal-Fetal Medicine, Department of Obstetrics and Gynecology, Indiana University School of Medicine, 975 West Walnut Street, IB 130, Indianapolis, IN 46202, USA

## Abstract

*Background*. As more women with metabolic muscle diseases reach reproductive age, knowledge of these diseases and their impact on pregnancy is necessary. *Case*. 23-year-old G1P0 with juvenile-onset Pompe disease (PD) delivered a viable infant by cesarean section at 32 weeks and 6 days. The pregnancy was complicated by worsening maternal pulmonary status, muscular strength, and mobility. *Conclusion*. The management of pregnancies complicated by Pompe disease requires a multidisciplinary approach, including expertise in neuromuscular disease, maternal-fetal medicine, biochemical genetics, pulmonology, anesthesia, and dietetics.

## Introduction

Pompe disease (PD), Type II glycogen storage disease, is a genetic disorder of glycogen degradation that is inherited in an autosomal-recessive pattern. It is caused by a deficiency of the enzyme acid *α*-glucosidase that degrades lysosomal bound glycogen to glucose. Glycogen accumulation results in lysosomal disruption and loss of muscle cell integrity. Patients with Pompe disease can develop progressive muscular weakness, cardiomyopathy, and respiratory failure, depending upon their age of onset [1]. The incidence of Pompe disease is widely variable based upon geography and ethnicity; however, the U.S. population incidence is 1 in 40,000 [2, 3].

The spectrum of disease includes infantile, juvenile-onset, and adult-onset forms. The infantile form presents within the first months of life with severe hypotonia, weakness, failure to thrive, feeding difficulties, cardiomyopathy, and progressive respiratory failure. Death frequently occurs within one year without treatment. Those with juvenile and adult onset disease can have onset of symptoms from infancy to late adulthood. Cardiac function is typically spared [1, 4]. The diagnosis may be made by measurement of enzyme activity in muscle or white blood cells or gene mutation analysis. Those with infantile onset demonstrate less than 1% of enzyme activity while those with juvenile- and adult-onset have less than 30% activity [1]. Mutations in the *GAA* (glucosidase, alpha, acid) gene, which produces the protein, lysosomal alpha-glucosidase, are associated with Pompe disease. The three most common mutations are p.Asp645Glu, p.Arg854X, and c.336-13T>G [4]. 

In 2006, the FDA approved Myozyme (alglucosidase alfa), a lysosomal glycogen-specific enzyme, as the first ever treatment for infantile-onset PD. In 2010, Lumizyme (alglucosidase alfa), was approved, extending treatment to patients aged 8 and older [5, 6]. As treatment is now available for Pompe disease and many other metabolic diseases, obstetricians must have knowledge about the pathophysiology of these disorders and their management during pregnancy. We present the complete management of pregnancy in a patient with Pompe disease.

## 2. Case

A 23-year-old G1P0 with juvenile-onset PD presented for prenatal care at 14-week and 4-day gestation. She had been diagnosed with PD 2 years prior via muscle biopsy and gene mutation analysis at another institution. The muscle biopsy revealed increased glycogen with decreased acid *α*-glucosidase activity of 0.38 *μ*mol/min/gm (low normal = 4.09, Athena Diagnostics [7]). Gene mutation analysis revealed two changes, c.1634C>T and c.2481+102_2646+31del. She described poor ambulation in childhood, with increasing weakness through adolescence and worsening pulmonary status over time. At her initial diagnosis of PD, her pulmonary function tests revealed an FEV1 (forced expiratory volume in 1 second) of 44% predicted and an FVC (forced vital capacity) of 43% predicted. 

Two months prior to presentation, the patient was seen by adult neurology to establish care. Based on FVC below 50% of predicted in a patient with neuromuscular disease, she was prescribed a noninvasive home ventilator (*BiPAP AVAP*). Shortly after the visit, but before acquiring the BiPAP, she learned she was 7 weeks pregnant. The patient immediately underwent repeat pulmonary function testing which revealed an FEV1 35% of predicted and FVC 36% of predicted in the sitting position with more than 25% drop in the supine position, indicative of severe restrictive pulmonary disease secondary to neuromuscular weakness. Arterial blood gas prior to and after BiPAP demonstrated *p*CO_2_ of 50 mmHg and 41 mmHg, respectively; therefore, BiPAP was instituted urgently. In addition, she was also immediately evaluated by titration polysomnography. Consultations by pulmonology, cardiology, and biochemical genetics were requested. 

At her initial prenatal visit, she was counseled on the risks of continuing the pregnancy including: chronic maternal hypoxia resulting in IUGR or IUFD; fetal hypoxia with subsequent long-term neurodevelopmental effects; worsening maternal pulmonary status and maternal death. The patient and her husband elected to continue the pregnancy. 

A high-protein (60–80 gm/day), low-carbohydrate diet and supplementation with levocarnitine (330 mg PO TID), coenzyme Q (100 mg PO TID), and ephedrine (25 mg PO TID) were initiated. At the time of the patient's pregnancy, enzyme replacement therapy was not available for adult use. The experimental use of enzyme replacement therapy was discussed, which the patient declined. 

Fetal well-being was monitored with serial growth ultrasounds. The patient underwent routine prenatal laboratory testing, all of which were within normal limits. The patient did not experience hypo- or hypertension, proteinuria, or hyperglycemia. 

Prior to pregnancy, she required a wheelchair intermittently; however, her weakness worsened throughout the pregnancy resulting in several falls. At 18-week gestation, she was not able to get out of a wheelchair unassisted. She subsequently required a powered mobility device and home health nursing for all activities of daily living. Throughout the pregnancy, the patient experienced persistent musculoskeletal pain, which was treated with hydrocodone, corticosteroid injections, lidoderm patches, and physical therapy. Due to the patient's decrease in mobilization and pregnancy-associated increased risk of thromboembolism, she was placed on Heparin for deep venous thrombosis (DVT) prophylaxis, 7500 units BID, which was increased to 10,000 units BID during the third trimester due to further decrease in activity. 

She was admitted at 30 weeks and 6 days after suffering a fall. She was administered a course of betamethasone. Worsening maternal respiratory and functional status led to the decision to keep the patient admitted. Due to poor oral intake, a nasojejunal (NJ) catheter was placed to administer supplemental nutrition. Peptamen AF was initiated with a goal rate of 65 mL/hr (1872 kcal, 17 gm protein). One week later, the patient developed increased work of breathing. Arterial blood gas revealed pH 7.456, *p*CO_2_ 39.1, and *p*O_2_ 89, and she was started on continuous BiPAP. 

At 33 weeks and 2 days, worsening maternal respiratory status prompted the need for delivery. Successful induction of labor was felt to be unlikely (Bishop score 1); therefore, cesarean section was recommended. After consultation with anesthesia, regional anesthesia was recommended because of the risk the patient might not be able to be extubated due to limited diaphragmatic and intercostal muscle strength. 

Preoperatively, the patient was initiated on total parenteral nutrition (TPN) as a bridge, while her tube feeds were held for six hours prior to surgery. In addition, prophylactic heparin was discontinued. The patient underwent cesarean section and tubal ligation under combined spinal-epidural anesthesia with delivery of a 3080 g infant. Apgars were 6 at one minute, 3 at 5 minutes, and 9 at 10 minutes. 

The patient was admitted to the intensive care unit (ICU) postoperatively as a precaution. In the immediate postoperative period, she was continued on total parental nutrition, enteral coenzyme Q, and intravenous L-carnitine; ephedrine was discontinued. NJ tube feeds were restarted postoperative day 1, and TPN was discontinued. Despite restarting heparin therapy, she developed an upper extremity DVT at her peripherally inserted central catheter (PICC) line site. She was initiated on therapeutic lovenox. The patient was discharged home on postoperative day 6. She was recommended to continue nightly NJ feeds until placement of a gastrostomy tube on postoperative day 17. At her two-month postpartum visit, she was continuing to use the gastrostomy tube and BiPAP at night. She was able to walk across her home occasionally. She did require increased assistance with mobility and activities of daily living compared to before pregnancy. Ten months after delivery pulmonary function returned to prepregnancy values with a FVC of 45% predicted and FEV1 of 42% predicted. One year after delivery, she began receiving intravenous enzyme replacement therapy (Lumizyme). She continues to require assistance with activities of daily living. She has antigravity strength in the proximal arms and requires a power chair. Her son has had normal development.

## 3. Comment

Pompe disease is an inherited genetic disorder of muscle metabolism with life expectancy dependent upon age of onset and degree of muscle involvement. Recent developments in diagnostic technology and treatment of Pompe disease have allowed more women to reach reproductive age, creating new management dilemmas for obstetricians. 

 Patients with Pompe disease must be strictly monitored for respiratory complications, metabolic derangements, and worsening functional status. With the additional physiological changes of pregnancy, these manifestations can significantly worsen. 

Patients with PD are often treated with a high-protein diet to avoid muscle wasting. Supplementation with coenzyme Q and L-carnitine have also been used to increase the metabolism of fat as an energy source, in an effort to decrease protein degradation and preserve muscle integrity [8, 9]. Ephedrine has been used to stimulate the degradation of glycogen, thereby decreasing toxic accumulation in lysosomes and limiting muscle damage during stress. 

Our patient was provided with a high-protein diet due to the presence of myopathy. Fasting was strictly avoided to prevent the storage of glycogen. Continuous NJ feeds with an elemental formula were initiated at 31-week gestation due to poor enteral intake and anticipated delivery. The stress of delivery, either by cesarean or vaginal, was felt to be a risk for increased protein degradation and worsening myopathy. Because our patient was recommended to undergo cesarean section, TPN was initiated preoperatively to provide continuous nutritional support for her muscles during surgery, as NJ feeds were discontinued 6 hours prior to surgery. 

Our patient was offered enzyme replacement therapy (ERT) as experimental treatment; however, it was declined due to unknown fetal and maternal risks. Recently, de Vries et al. reported on the continued use of Lumizyme (alglucosidase alfa) during pregnancy, and no fetal or maternal side effects were noted [10]. The patient described by de Vries was initiated on enzyme replacement therapy 17 months prior to pregnancy; however, our patient was not on therapy prior to pregnancy as the adult use of ERT had not been approved. Lumizyme is reported as a Category B drug. Mice and rabbit studies have revealed “no impaired fertility or harm to the fetus” using a dose of 40 mg/kg/day (0.4 to 0.5 times the human steady-state exposure) [5]. Unfortunately, no controlled human studies have been performed. Due to the large volume of enzyme required to achieve correct dosing, we also had a concern regarding possible fluid overload in a patient with compromised pulmonary function. However, de Vries reported no worsening of maternal symptoms with enzyme administration [10]. Obstetricians will need to review the possible risks and benefits of ERT with patients and determine appropriateness of its' use on an individual basis. 

Pregnancy is commonly associated with respiratory alkalosis. Due to our patient's neuromuscular weakness, treatment goals were to achieve near-normal pH and *p*CO_2_ values. With the progression of increasing tidal volume and increasing intercostal and diaphragmatic weakness, our patient required nightly and subsequently continuous BiPAP. 

Interestingly, the infant weight was noted to be greater than the 97th percentile for gestational age. Antenatal screening for gestational diabetes was negative. Our patient gained 6.9 kg (15.2 pounds) during the pregnancy. With a prepregnancy body mass index (calculated as weight (kg)/[height (m)]^2^) of 25.6, the patient's weight gain was appropriate per ACOG recommendations [11]. One possible explanation for an infant fetal weight > 97th percentile is the maternal diet of high-protein and high-calorie foods. 

The recurrence risk for Pompe disease is dependent on paternal carrier status. With an incidence of 1 in 40,000 in the USA, the carrier rate for Pompe disease is 1 in 100. If paternal carrier status is unknown, the risk of having an affected child is 1 in 200 (Hardy-Weinberg law [12]). Prenatal testing for Pompe disease is available by chorionic villus sampling or amniocentesis. If familial mutations have been identified, molecular genetic testing is preferred. Otherwise, enzymatic assay on amniocytes should be performed. 

This case demonstrates that a multidisciplinary approach of obstetric care for patients affected by Pompe disease can lead to a successful pregnancy outcome and serve as a bridge to enzymatic therapy. It should be noted, however, that our patient did have a significant decline in her muscle strength during the pregnancy, which has not been recovered despite enzyme replacement and supportive nutritional therapy. Although the continued use of ERT during pregnancy has been reported, the initiation of ERT during pregnancy has not. Women with Pompe disease should seek preconceptional counseling to discuss the use of enzyme replacement therapy during pregnancy, as well as, the impact of pregnancy on disease progression.
